# The effect of gender-role orientation on attitudes towards menstruation in a sample of female university students

**DOI:** 10.4274/jtgga.galenos.2018.2018.0122

**Published:** 2019-08-28

**Authors:** Ashraf Ghiasİ

**Affiliations:** 1 Student Research Committee, School of Nursing and Midwifery, Shahroud University of Medical Sciences, Shahroud, Iran

**Keywords:** Attitudes, Bem Sex Role Inventory, female students, gender-role orientation, menstruation

## Abstract

**Objective:**

To examine the effect of gender role orientation on attitudes towards menstruation in a sample of Iranian female students of medical sciences.

**Material and Methods:**

Three hundred female university students (94%; response rate: 282) were enrolled in the study via stratified random sampling. Data were collected using a demographic questionnaire, the Menstrual Attitude Questionnaire (MAQ), and the short version of the Bem Sex Role Inventory (BSRI). Data were analyzed using SPSS v.18. Analyses were performed using the Kruskal-Wallis test and the Mann-Whitney U test.

**Results:**

The mean scores of the MAQ subscales ranged from 3.7±1.35 to 5.6±1.3, indicating that most of the respondents had natural to moderate attitudes toward menstruation. When participants were classified into one of four gender-role categories of BSRI, the results showed that the undifferentiated group with 33.7% was higher than other gender-role groups. The undifferentiated group was significantly less likely than the other groups to perceive “menstruation as a natural event”.

**Conclusion:**

The study shows an association between gender-role orientation and attitudes toward menstruation in female university students. However, further research is still necessary in this issue.

## Introduction

Menstruation, the cyclical shedding of blood and endometrium from the uterine cavity, is a physiologic process that occurs throughout a woman's reproductive years (1). Although menstruation is a natural/biologic event, perimenstrual symptoms (immediately before and during menstruation), including anxiety, depression, irritability, tension, mood swings, fatigue, skin disorders, breast tenderness, swelling, weight gain, cramps, and backache affect a significant percentage of women (2,3). Evidence suggests that attitudes toward menstruation can influence the reporting of perimenstrual symptoms (4). For example, Lu (5) found a significant association between negative attitudes toward menstruation and the experience of perimenstrual symptoms in Taiwanese women. Studies have also demonstrated that a woman’s beliefs about and attitudes toward menstruation were influenced by socio-cultural factors and family environments (6,7,8). For example, Hoerster et al. (9) compared Indian and American women’s attitudes toward menstruation. They found that menstruation was perceived as significantly more debilitating and a less natural event by American women compared with Indian women (9). A few studies investigated the effect of gender-role orientation – the extent to which a person believes or perceives that she/he possesses gender-typed characteristics – on attitudes toward menstruation (10,11). Chrisler (11) showed that undifferentiated and feminine college students were more likely than androgynous and masculine students to perceive menstruation as a bothersome event; undifferentiated and masculine college students were more likely than androgynous and feminine students to perceive menstruation as a debilitating event. The effect of gender-role orientation on menstrual attitudes is not entirely clear. Hence, in the present study, the Menstrual Attitude Questionnaire (MAQ) and Bem Sex Role Inventory (BSRI) were administered to a sample of female students with the aim of examining the impact of gender-role orientation on attitudes toward menstruation.

## Material and Methods

### Participants

In the academic year 2015/16, there were nearly 900 female students at 4 schools of Shahroud University of Medical Sciences. Thus, the sample size was estimated as 269 using a Krejcie & Morgan table. After adding a 10% non-response rate, the final sample size for this cross-sectional study became 300. Stratified random sampling was used to choose the study participants. The inclusion criteria in this study were as follows: Iranian nationality, aged between 18 and 30 years, and no history of polycystic ovary syndrome or mental disorders.

### Instruments

Data were collected using a demographic questionnaire, the 30-item version of the BSRI, and the MAQ.

The demographic questionnaire included questions about menstrual status (age at first menstruation, menstrual cycle length, menstrual frequency, and regulation of menstruation), age, and marital status.

The original (BSRI Bem, 1974) includes 20 masculine, 20 feminine, and 20 neutral items, each item ranges from 1 ‘never/almost never true’ to 7 ‘always/almost always true’. It was designed to categorize subjects into four groups: Masculine (high masculine, low feminine), feminine (high feminine, low masculine), androgynous (high masculine, high feminine), and undifferentiated (low masculine, low feminine) (12). In this study, the short 30-item version of the BSRI was used. The validity and reliability of the Persian version of this questionnaire were confirmed in previous studies (13). In the present study, the internal consistency coefficient of the femininity and masculinity subscales were 0.76 and 0.84, respectively.

The MAQ comprises 33-items divided into five subscales: (1) menstruation as a deliberating event (12 items), (2) menstruation as a bothersome event (6 items), (3) menstruation as a natural event (4 items), (4) anticipation and prediction of the onset of menstruation (4 items), and (5) denial of any effects of menstruation (7 items). The items are scored on a Likert scale (1: strongly disagree to 7: strongly agree) (14). In the current study, the alpha coefficient values of the five subscales ranged between 0.77 and 0.85.

### Statistical analysis

Data were analyzed using SPSS v.18. Descriptive statistics were calculated where appropriate for each variable. The Kruskal-Wallis test and Mann-Whitney U test were used to examine the impact of gender-role orientation on the female university students’ attitudes toward menstruation. P<0.05 was considered statistically significant.

## Results

Eighteen recruited participants for the study were excluded because of failure to complete the questionnaire, resulting in a response rate of 94%. The participants' mean age was 21.8 (±2.2) years. The mean age at onset of menstruation was 12.81 (±1.49) years, the mean length of menstrual cycle was 6.43 (±1.39) days, and the mean menstrual frequency was 28.87 (±4.4) days. Most (71.6%) study participants had a regular menstrual pattern and the majority (88.3%) was single.

The mean scores on the MAQ subscales ranged from 3.7±1.35 to 5.6±1.3, indicating that most of the participants had natural to moderate attitudes toward menstruation (Table 1).

In order to determine the gender roles of feminine, masculine, androgynous and undifferentiated, masculine and feminine median scores were calculated. Median masculinity score was M: 5.36 and the median femininity score was F: 5.6.

In this study, 16.6% (n=47) of the participants were in the feminine gender role group, 16.6% (n=47) were masculine, 33.7% (n=95) were undifferentiated, and 33% (n=93) of the participants were in the androgynous gender role group.

There was a significant difference in the “menstruation as a natural event” subscale of the MAQ among the participants based on the BSRI – masculine, feminine, undifferentiated, and androgynous – (p<0.05) (Table 2).

As seen in Table 3, the undifferentiated group was significantly (p<0.05) less likely to perceive menstruation as a natural event than the androgynous, feminine, and masculine groups.

## Discussion

The current study investigated the effect of gender-role orientation on attitudes toward menstruation in a sample of female university students. When analyzing attitudes toward menstruation, the results showed that the highest and lowest mean scores on the MAQ subscales among the participants were the anticipation and prediction of the onset of menstruation and the denial of any effects of menstruation, respectively. This result is consistent with a previous study among women in the United States military (15). In another study by Guvenc et al. (16) among Turkish nursing students, the highest and lowest mean scores on the MAQ subscales were menstruation as a natural event and denial of any effects of menstruation, respectively. When participants were classified into one of four gender-role categories of BSRI, masculine, feminine, androgynous, or undifferentiated, results show that the percentage of the undifferentiated group was higher than other gender-role groups.

In a study by Mullis and McKinley (10), masculine was the most frequent gender-role type among a sample of female adolescents. These differences between studies could be due to different cultural, social, or religious backgrounds (13,17). In the present study, there was a significant difference in only one of the five subscales of the MAQ based on four gender-role categories of BSRI. Undifferentiated individuals were significantly less likely to perceive menstruation as a natural event than the other gender role types. This indicates that gender-role orientation is a small to moderate contributor to women's attitude toward menstruation.

A previous study by Chrisler (11) was conducted on two samples. Sample A included 11 men, aged 28-39 years, and 20 women, aged 30-45 years. Sample B comprised 19 men, aged 18-22 years, and 37 women, aged 18-23 years. The results showed that in sample A, gender orientation had no significant effect on attitudes toward menstruation. However, in sample B, undifferentiated and feminine college students were more likely to perceive menstruation as a bothersome event than the androgynous and masculine students; undifferentiated and masculine college students were more likely to perceive menstruation as a debilitating event than the androgynous and feminine students.

Several limitations in the study ought to be considered. This research was conducted among female students of medical sciences; the findings may not be same for other segments of the female population. Also, because the study has a cross-sectional design, it can only illuminate the current situation of the participants. Furthermore, this study relied on self-reports of gender-role orientation, and these reports may not have always been accurate.

In conclusion, in this study there was a significant difference in the “menstruation as a natural event” subscale of the MAQ among female university students based on four categories of BSRI (androgynous, undifferentiated, masculine, and feminine). The undifferentiated group was significantly less likely to perceive menstruation as a natural event than the other groups.

## Figures and Tables

**Table 1:**
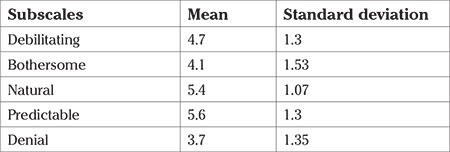
Mean and standard deviation scores on the subscales of menstrual attitude questionnaire

**Table 2:**
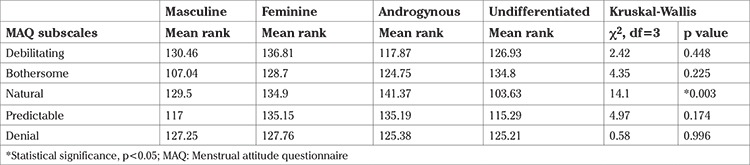
Differences in attitudes toward menstruation based on gender role orientation

**Table 3:**
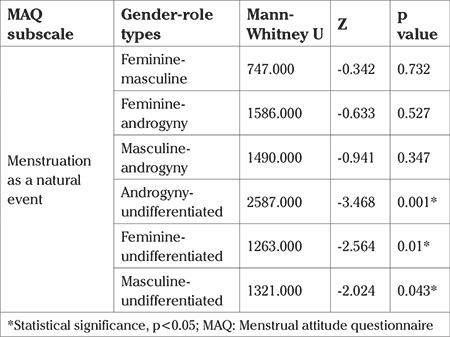
The Post-Hoc Mann-Whitney U test results
